# Seropositive Rubella and Toxoplasmosis Among Females Attending Saudi Hospital

**DOI:** 10.1155/jotm/4201991

**Published:** 2025-02-13

**Authors:** Nabeel H. Alhussainy, Faten A. Al Braikan

**Affiliations:** Department of Clinical Microbiology and Immunology, Faculty of Medicine, King Abdulaziz University, Jeddah 21589, Saudi Arabia

**Keywords:** IgG, IgM, rubella, Saudi hospital, toxoplasmosis

## Abstract

**Background:** *Toxoplasma gondii* and rubella virus are significant health concerns among women. Therefore, it is crucial to determine the prevalence of their antibodies.

**Objective: **This study aimed to explore the prevalence of rubella and toxoplasmosis immunoglobulin G (IgG) antibodies among females who attended different clinics in King Abdulaziz University Hospital, Jeddah, Saudi Arabia.

**Method:** A retrospective observational study was conducted among nonpregnant women who attended various clinics at King Abdulaziz University Hospital in Jeddah, Saudi Arabia. The study included 540 female participants who visited various clinics, with a mean (standard deviation [SD]) age of 31.92 (6.175) years and a median (interquartile range [IQR]) of 32 (8). These women were tested for rubella and toxoplasmosis IgG and immunoglobulin M (IgM) from January 2021 to June 2022. ELISA test for detecting antitoxoplasmosis and antirubella IgG and IgM antibodies was conducted using kits manufactured by Abbott, located at Max-Planck-Ring 2, 65,205 Wiesbaden, Germany.

**Results:** The majority of the participants were from Saudi Arabia (78.1%). Most females (73.3%) had positive results for rubella IgG, while only 5.6% tested positive for toxoplasmosis IgG. A significantly higher percentage of positive rubella-G antibodies was seen between those with positive toxoplasmosis IgG and those with negative toxoplasmosis IgG (93.3% vs. 76.4%, *p*=0.032). Non-Saudi females had a significantly higher rate of positive toxoplasmosis IgG than Saudi women (15.4% vs. 2.9%, *p* < 0.001).

**Conclusion:** The study revealed a high prevalence of rubella antibodies and a low prevalence of toxoplasmosis antibodies among females living in Saudi Arabia, with a higher prevalence of toxoplasmosis antibodies seen among non-Saudi females. A significant association between the prevalence of rubella and toxoplasmosis antibodies was found. Therefore, raising awareness about the risks and prevention measures of toxoplasmosis is crucial, emphasizing hygiene practices.

## 1. Introduction

The causative agent of toxoplasmosis is *Toxoplasma gondii*, an intracellular obligate parasite that causes abortion in humans and animals [1]. Lymphadenitis is the most common clinical form of the disease [2]. The parasite does not spread directly from person to person. Intermediate hosts can become infected through various means, such as contaminated water or food [3]. The diagnosis of toxoplasmosis still depends on detecting different types of antibodies, each with a different interpretation. If immunoglobulin M (IgM) is detected in the patient's blood sample using the enzyme-linked immunosorbent assay (ELISA) technique, it indicates an infection in the acute phase, while positive IgG indicates a past infection [4].

The World Health Organization has confirmed that approximately 30%–50% of people worldwide have been infected with *Toxoplasma gondii* [5]. Over one-third of the world's human population is infected with *Toxoplasma gondii*, and they do not show any symptoms because their immune system usually prevents the parasite from causing illness. A chronic, usually lifelong, infection with *Toxoplasma* that does not exhibit clinical symptoms of toxoplasmosis disease is called latent toxoplasmosis. On the other hand, chronic infection associated with continuous or recurrent clinical symptoms is termed as chronic toxoplasmosis [6].

The rubella virus was initially isolated from cell culture in 1962 [7]. The rubella virus, belonging to the Togaviridae family (genus *Rubivirus*), is an enveloped, single-stranded RNA virus that infects humans and is transmitted through the respiratory route [8]. The rubella virus, also known as German measles due to its discovery by German physicians, causes a self-limiting viral infection commonly seen in children worldwide. It leads to fever and a rash similar to that of measles [9].

Before 1969, there were several reports of rubella outbreaks in children under 12 years before the vaccine's introduction [10]. Due to vaccination programs, the number of rubella infections has decreased significantly [9]. The measles, mumps, and rubella (MMR) vaccine has been available in India since 2000. However, the rubella-containing vaccine was only introduced in the National Immunization Schedule in 2017 [11].

Concurrent infection with *Toxoplasma gondii* and the rubella virus during pregnancy significantly amplifies the risk of severe adverse outcomes for both the mother and the fetus. Dual infections can have a compounding effect, increasing the likelihood of vertical transmission and exacerbating the severity of congenital anomalies. *T. gondii* infection may result in complications such as miscarriage, stillbirth, or profound neurological and ocular damage [5], while rubella infection, particularly in the first trimester, can cause congenital rubella syndrome (CRS), characterized by congenital heart defects, cataracts, microcephaly, and intellectual disability [9], When these infections coexist, the fetus is at an even greater risk of developmental disruption due to overlapping pathological mechanisms, including widespread inflammation, immune dysregulation, and direct tissue damage. Early detection through prenatal screening, coupled with immediate medical intervention, is essential to manage and mitigate the compounded risks associated with dual infections. Preventive measures, including rubella vaccination before conception and practices to prevent toxoplasmosis, remain the cornerstone of reducing the incidence and impact of such coinfections [12], It is crucial to raise awareness about vaccination to prevent toxoplasmosis and rubella viruses. In addition, screening for rubella and toxoplasmosis IgG antibodies will enhance public health. Therefore, our study aimed to evaluate the prevalence of rubella and toxoplasmosis IgG antibodies among females who visited various clinics at King Abdulaziz University Hospital, Jeddah, Saudi Arabia.

## 2. Methodology

This retrospective observational study was performed among women who attended different clinics at King Abdulaziz University Hospital, Jeddah, Saudi Arabia, from January 2021 to June 2022. The study included nonpregnant women who had available rubella and toxoplasmosis IgG test results.

Data were extracted from electronic medical records of women who attended different clinics at King Abdulaziz University Hospital, Jeddah, Saudi Arabia. The collected data included the participants' characteristics, such as age, nationality, and reason for visiting the clinic. The results of rubella and toxoplasmosis IgG and IgM tests were also collected. ELISA tests for the detection of antitoxoplasmosis and antirubella IgG and IgM antibodies were conducted using kits manufactured by Abbott, located at Max-Planck-Ring 2, 65205 Wiesbaden, Germany, with a relative specificity of 99.89%

Approval was given by the Research and Ethical Committee of the King Abdulaziz Scientific Research Ethics Committee with ethics number: 654-23 on 28 November 2023. Confidentiality was maintained through all research steps.

Statistical analysis was conducted using the IBM SPSS computer program (Version 26.0, Armonk, New York, United States of America). Categorical variables were expressed in frequencies and percentages. Continuous, non-normally distributed variables were reported as the mean ± standard deviation, median and interquartile range (IQR), and minimum and maximum values. The chi-square test was used to compare categorical variables. The statistical significance was established by considering *p* values below 0.05.

## 3. Results

The study was conducted among 540 nonpregnant females who attended different Clinics at King Abdulaziz University Hospital, Jeddah, Saudi Arabia, from January 2021 to June 2022. Their mean (SD) age was 31.92 (6.175) years. The majority were Saudis (78.1%). The majority of females (73.3%) had positive rubella-G, and only 5.6% had positive Toxo-G, as shown in Table 1.

Table 2 illustrates the chief complaints among female participants. Laboratory examinations (17.0%) and diabetes mellitus (8.5%) were the most common reasons for visiting clinics.

There was a significant association between having rubella and toxoplasmosis IgG antibodies (*p*=0.032). The percentage of females with positive rubella-G was higher in those with positive Toxo-G (93.3%) than in negative Toxo-G antibodies (76.4%). However, there was no significant association between female participants' demographics and having positive rubella-G. Meanwhile, nationality (*p* < 0.001) was the only significant factor affecting seropositivity of toxoplasmosis with IgG. Non-Saudi females (15.4%) had a higher percentage of seropositivity of Toxo-G than Saudi women (2.9%), as shown in Table 3.

## 4. Discussion

Our study aimed to assess the prevalence of having positive rubella and toxoplasmosis IgG antibodies among nonpregnant females who attended different clinics at King Abdulaziz University Hospital, Jeddah, Saudi Arabia. The findings of our study reveal a high prevalence of rubella antibodies and a low prevalence of toxoplasmosis antibodies among participants. This high prevalence of rubella antibodies likely reflects successful immunization efforts or past exposure leading to immunity. Conversely, the low prevalence of toxoplasmosis antibodies may indicate either limited exposure to the parasite or effective preventive measures within the population. The significant association observed between positive rubella and toxoplasmosis antibodies suggests potential shared risk factors or immune responses that warrant further investigation.

This study revealed a high prevalence (73.3%) of rubella IgG antibodies and a low prevalence (5.6%) of toxoplasmosis IgG antibodies among females. Furthermore, the study revealed a significantly higher positive toxoplasmosis IgG rate among non-Saudi females. This higher prevalence may be attributed to the fact that non-Saudi females are more likely to come from regions where toxoplasmosis is more common or where public health measures to control the disease are less. A comprehensive review reveals that the global seroprevalence of *Toxoplasma gondii* infection can range from as low as 0.5% to as high as 87.7%. Notably, areas such as Africa (61.4%), Oceania (38.5%), and South America (31.2%) report higher infection rates. In contrast, regions in Asia, including Saudi Arabia, typically experience lower rates of infection. Several factors contribute to these differences, including variations in dietary practices, hygiene standards, and public health initiatives. For instance, places with elevated infection rates often see a higher consumption of undercooked meat and a greater likelihood of exposure to contaminated soil and water [12].

The prevalence of toxoplasmosis IgG antibodies revealed in our study is considered low compared to the findings of a systematic review conducted in 2020, which indicates that toxoplasmosis is a widespread infection among humans and animals in Saudi Arabia, with prevalence rates varying significantly based on geographical location, population demographics, and specific exposure risks [13].

A study examined the seropositivity of toxoplasmosis IgG among the general population in Qatar and found a seropositivity rate of 29.8% [14]. Another study in the United Arab Emirates (UAE) revealed a comparable percentage of toxoplasmosis IgG seropositivity (22.9%) [15]. In Kuwait, the seropositivity rate of toxoplasmosis IgG was higher than in Qatar and UAE (45.7%) [16]. In Iran, 73.8% had IgG anti-*Toxoplasma gondii* antibodies [17].

Previous literature found that Saudi Arabia had a lower prevalence of toxoplasmosis IgG than other countries. A study in Jeddah found that 4.7% of university students had *Toxoplasma gondii* IgG antibodies, which aligned with our results [18]. A study published in Tabuk also found that 9.4% of female University students were seropositive for *Toxoplasma gondii* IgG antibodies [19]. Another study in Makkah found a 19.5% prevalence rate of toxoplasmosis IgG antibodies among blood donors [20]. This could justify the significantly higher prevalence of toxoplasmosis IgG among non-Saudi in our study compared to Saudi females. Moreover, these variations may be attributed to human factors such as diet, social and economic patterns, cultural influences, local water quality, and access to proper sanitation [21]. Examining these potentially influential factors is crucial, as *Toxoplasma gondii* has a significant adverse impact on human health and can lead to severe outcomes in immunocompromised patients [22].

In addition, our study found a significant association between seropositivity of rubella IgG antibodies and toxoplasmosis IgG antibodies. However, the literature regarding this relationship was limited. This association could return to other confounding factors, such as vaccination of rubella and toxoplasmosis IgG among positive females.

Concerning rubella IgG, our study found that 73.3% of females had positive rubella IgG antibodies. Similarly, a study in South India found that 68.3% of girls aged 13–15 had positive rubella IgG antibodies [23]. Another study in North India found that the seroprevalence rate of rubella IgG antibodies was 67.3% among girls aged 11–18 years [24]. However, other studies showed a higher prevalence than our study. A study in Turkey showed that seroprevalence of rubella-specific IgG antibodies was 92.5% among schoolgirls within the age range of 12–18 years [25]. Another study in Zambia found seropositivity of 91.9% of rubella IgG antibodies among female blood donors [26].

The higher prevalence of rubella compared to toxoplasmosis in Saudi Arabia can be attributed to several factors. First, the country has implemented widespread rubella vaccination programs, particularly targeting schoolgirls and women of childbearing age, which has significantly increased immunity against rubella in the population. In contrast, toxoplasmosis is typically contracted through the consumption of undercooked meat or exposure to contaminated water and soil. Public health measures specifically aimed at controlling toxoplasmosis may not be as comprehensive [27]. In addition, cultural dietary practices and food safety standards may vary between regions, affecting the rate of exposure to *Toxoplasma gondii*. Rubella, being a viral infection, spreads more easily through respiratory droplets, while toxoplasmosis requires specific conditions for transmission. These factors collectively help explain the higher prevalence of rubella compared to toxoplasmosis in Saudi Arabia [28].

Therefore, it is important to strengthen rubella vaccination efforts and raise awareness about toxoplasmosis prevention. Besides, improving diagnostic capabilities for toxoplasmosis and providing preconception counseling can protect females from potential risks associated with these infections.

### 4.1. Limitation

The current study's retrospective observational design limits our findings to adjusting for other potential confounding variables that may influence the results. Therefore, it is highly recommended that a large trial be conducted with a more comprehensive investigation of all possible factors.

## 5. Conclusion

The study revealed a high prevalence of rubella antibodies and a low prevalence of toxoplasmosis antibodies among participants. A significant association between positive antibodies for both infections suggests that exposure to one may increase the likelihood of exposure to the other due to shared risk factors or immune responses. Raising awareness about the risks of rubella and toxoplasmosis, especially among females, and promoting hygiene practices are crucial. The high rubella antibody prevalence may indicate past infection or successful immunization, while the low toxoplasmosis antibody prevalence could reflect lower exposure or effective prevention. Further research is essential to monitor infection prevalence, identify risk factors, and assess preventive measures, which can enhance public health strategies and mitigate the impact of these infections.

## Figures and Tables

**Table 1:** Baseline characteristics of the participated women (*N* = 540).

Age (years)	Mean (SD)	31.92 (6.175)	
	Median (IQR)	32 (8)	
	Min-max	8–64	

**Parameters**	**Category**	**Number**	**Percentage**

Nationality	Saudi	422	78.1
	Yemeni	30	5.6
	Indian	42	7.8
	Egyptian	13	2.4
	Other	33	6.1

Toxoplasmosis IgG	Negative	506	93.7
	Equivocal	4	0.7
	Positive	30	5.6

Toxoplasmosis IgM (*N* = 91)	Negative	90	98.9
	Gray zone	1	1.1

Rubella IgG (*N* = 539)	Negative	116	21.5
	Equivocal	27	5.0
	Positive	396	73.3

Rubella IgM (*N* = 19)	Negative	19	100.0

Rubella IgM (*N* = 0)	Positive	0	0

**Table 2:** Chief complaints for female participants (*N* = 519).

Parameters	Category	Number	Percentage
Chief complaint	Laboratory examination	88	17.0
	Diabetes mellitus	44	8.5
	Hypothyroidism	28	5.4
	Abdominal pain	21	4.0
	Pain	20	3.9
	Iron deficiency	19	3.7
	General medical examination	19	3.7
	Obesity	18	3.5
	Acute urinary tract infection	16	3.1
	Other⁣^∗^	262	50.1

⁣^∗^Fever, headache, gastritis, chest pain, lower back pain, fatigue, pain in the joint, hypertension, asthma, leg pain, cough, systemic lupus erythematosus, lump in breast/fibroadenoma of breast, dizziness, urticaria, allergic rhinitis, allergy, HIV, hyperprolactinemia, acne vulgaris, acute pharyngitis, gastroenteritis and colitis, nausea and vomiting, calculus of gallbladder with other cholecystitis, dyspepsia, malignant neoplasm of the thyroid gland, localized swelling, otitis media, urinary incontinence, viral pharyngoconjunctival fever, anxiety disorder, nonscarring hair loss, special screening exam, beta thalassemia, candidiasis, sepsis, telogen effluvium, constipation, covid, cutaneous mycobacterial infection, deviated nasal septum, renal colic, rheumatoid arthritis, retention of urine, other atopic dermatitis, other chronic renal failure, other chronic sinusitis, and nephrotic syndrome diffuse membranous glomerulonephritis.

**Table 3:** Association between the presence of immunoglobulin G of rubella and toxoplasmosis and demographics.

Parameters	Category	Rubella IgG *N* (%)		*p* value
		Positive	Negative	
Age (years)	Less than or equal to 30	159 (73.3)	58 (26.7)	0.059
	More than 30	237 (80.3)	58 (19.7)	

Nationality	Saudi	306 (76.9)	92 (23.1)	0.643
	Non-Saudi	90 (78.9)	24 (21.1)	

Toxoplasmosis IgG	Positive	28 (93.3)	2 (6.7)	0.032
	Negative	366 (76.4)	113 (23.6)	

**Parameters**	**Category**	**Toxoplasmosis IgG N (%)**		**p** **-value**
		**Positive**	**Negative**	

Age (years)	Less than or equal to 30	12 (5.2)	218 (94.8)	0.740
	More than 30	18 (5.9)	288 (94.1)	

Nationality	Saudi	12 (2.9)	407 (97.1)	< 0.001
	Non-Saudi	18 (15.4)	99 (84.6)	

## Data Availability

The data that support the findings of this study are available from the corresponding author upon reasonable request. Restrictions may apply to the availability of certain data, which were used under license for the current study, and are therefore not publicly available. However, data are available from the authors upon reasonable request and with permission from the appropriate parties.
